# Decreased Levels of Active SMAD2 Correlate with Poor Prognosis in Gastric Cancer

**DOI:** 10.1371/journal.pone.0035684

**Published:** 2012-04-23

**Authors:** Yijun Wu, Qi Li, Xinhui Zhou, Jiren Yu, Yunchuan Mu, Stefan Munker, Chengfu Xu, Zhe Shen, Roman Müllenbach, Yan Liu, Li Li, Norbert Gretz, Derek Zieker, Jun Li, Kouichi Matsuzaki, Youming Li, Steven Dooley, Honglei Weng

**Affiliations:** 1 Department of General Surgery, The First Affiliated Hospital, Zhejiang University School of Medicine, Hangzhou, China; 2 Molecular Hepatology - Alcohol Associated Diseases, II, Medical Clinic Faculty of Medicine at Mannheim, University of Heidelberg, Mannheim, Germany; 3 Department of Gynecology, The First Affiliated Hospital, Zhejiang University School of Medicine, Hangzhou, China; 4 Department of Gastroenterology, The First Affiliated Hospital, Zhejiang University School of Medicine, Hangzhou, China; 5 Department of Medicine II, Saarland University Hospital, Saarland University, Homburg, Germany; 6 Medical Research Center, Medical Faculty Mannheim, University of Heidelberg, Mannheim, Germany; 7 General, Visceral Surgery and Transplantation, University Hospital Tübingen, Tübingen, Germany; 8 Departments of Gastroenterology and Hepatology, Kansai Medical University, Osaka, Japan; University of Navarra, Spain

## Abstract

**Background:**

TGF-β plays a dual role in the progression of human cancer. During the early stages of carcinogenesis, TGF-β functions as a tumor suppressor. During the late stages of tumor development, however, TGF-β can promote tumor growth and metastasis. A shift in Smad2/3 phosphorylation from the carboxy terminus to linker sites is a key event determining biological function of TGF-β in colorectal and hepatocellular carcinoma. In the present study, we investigated the potential role of differential Smad2/3 phosphorylation in gastric adenocarcinoma.

**Methodology:**

Immunohistochemical staining with anti-P-Smad2/3C and P-Smad2/3L antibodies was performed on 130 paraffin-embedded gastric adenocarcinoma specimens. The relationship between P-Smad2/3C and P-Smad2/3L immunohistochemical score and clinicopathologic characteristics of patients was analyzed. Real time PCR was used to measure mRNA expression of Smad2 and Smad3 in cancer and surrounding non-tumor tissue.

**Principal Findings:**

No significant P-Smad2L and/or P-Smad3L positive staining was detected in the majority of specimens (positive staining in 18/130 samples). Positive P-Smad2/3L staining was not associated with a decrease in carboxyterminal phosphorylation staining. Loss of P-Smad2C remarkably correlated with depth of tumor infiltration and poor differentiation of cancer cells in patients with gastric cancer. No correlation was detectable between P-Smad3C and clinicopathologic characteristics of gastric adenocarcinoma. However, co-staining analysis revealed that P-Smad3C co-localised with α-SMA and collagen I in gastric cancer cells, indicating a potential link between P-Smad3C and epithelial-to-mesenchymal transition of cancer. Real time PCR demonstrated reduced mRNA expression of Smad2 in gastric cancer when compared with surrounding non-tumor tissue in 15/16 patients.

**Conclusions:**

Loss of P-Smad2C tightly correlated with cancer invasion and poor differentiation in gastric cancer. Contrary to colorectal and hepatocellular carcinoma, canonical carboxy-terminal phosphorylation, but not linker phosphorylation, of Smad2 is critical for gastric cancer.

## Introduction

Gastric cancer is a leading cause of cancer-related death in the world, ranking second in males and fourth in females in frequency [1]. The pathogenesis of gastric cancer is associated with multiple factors. Among these, dysregulation of signaling pathways related to developmental processes, including transforming growth factor-β (TGF-β), Wnt/β-catenin, hedgehog and Notch signaling, plays a central role in development and progression of this cancer [2].

The TGF-β family of molecules, including TGF-β isoforms, activins and bone morphogenetic proteins (BMPs), has important functions in various physiological and pathophysiological processes, e.g. embryonic development, autoimmune diseases, fibrosis and cancer [3], [4]. TGF-β transduces its signals by stimulating formation of heteromeric complexes of TGF-β type I (TGF-βRI) and type II (TGF-βRII) serine/threonine kinase receptors. Activated TGF-βRI propagates signaling by recruitment and phosphorylation of receptor-regulated-Smads (R-Smads, including Smad2 and Smad3). Phosphorylation of C-terminal serine residues in R-Smads is a crucial step for canonical TGF-β signaling. The two most C-terminal serine residues at serine 465/467 in Smad2 and serine 423/425 in Smad3 are phosphorylated, together with a third non-phosphorylated serine residue, form an evolutionarily conserved SSXS motif in all R-Smads [5], [6]. Besides C-terminal phosphorylation of Smad2/3 (P-Smad2C and P-Smad3C) by TGF-βRI, other kinases, e.g. c-Jun N-terminal kinase (JNK) and Ras-associated kinases, cause phosphorylation of R-Smads at linker sites around serine 249/254 in Smad2 and serine 208/213 in Smad3 (P-Smad2L and P-Smad3L) [7], [8]. Phosphorylated R-Smads form a complex with common Smad (Co-Smad; Smad4 in mammals) and shuttle into the nucleus for target gene transcription [3]. Besides R-Smad and co-Smad, the third type of Smad protein is inhibitor-Smad (I-Smad; Smad6 and Smad7). I-Smads are transcriptionally induced by TGF-β, indicating a negative feedback mechanism of this signaling pathway [4].

TGF-β plays a dual role in the progression of human cancer [9], [10]. In the early stages of cancer, TGF-β acts as a tumor suppressor by inhibiting cellular proliferation or by promoting cellular apoptosis. However, in the late stages, TGF-β supports tumor progression such as tumor cell invasion, dissemination and immune evasion [9]. In addition, TGF-β is well recognised as a mediator of epithelial-to-mesenchymal transition (EMT) in cancer [11]. Although perturbations of TGF-β/Smad signaling are central to carcinogenesis in most of organs, its tumor promoting outcome is highly context-dependent. For example, TGF-β signaling is pivotal in the maintenance of cancer stem cell self-renewal and tumorigenic activity in glioma and leukaemia, whereas the effects of TGF-β signaling in breast cancer stem cell are controversial [12]. One study showed that blocking TGF-β pathway via a dominant negative TGF-βRII increases the size of breast stem cell compartment and promotes tumorigenesis, indicating a suppression of breast carcinogenesis of this cytokine [13]. By contrast, Mani and colleagues found that TGF-β pathway us critical in the maintenance of breast cancer stem cell-like properties and tumorigenic activity via inducing EMT [14].

In gastric cancer, single-nucleotide polymorphisms (SNPs) of TGF-β are associated with susceptibility to stage I and stage II of gastric cancer [15], [16]. The serum levels of TGF-β were reported to significantly correlate with venous invasion in patients with gastric cancer [17]. However, detailed mechanisms of TGF-β signaling in gastric cancer progression are still unknown. In addition, it remains unclear when and how TGF-β transforms from a tumor suppressor into a tumor promoter during carcinogenesis.

Recently, a shift in phosphorylation of Smad2/3 from C-terminal to linker sites was demonstrated as a key event determining biological function of TGF-β in cancer [18], [19], [20]. Previous studies based on 12 year follow-up demonstrated that chronic HBV/HCV patients with P-Smad2/3L in liver tissues had a higher risk of advancing to hepatocellular carcinoma (HCC) than those with P-Smad2/3C [8], [21]. Consistent with the findings in HCC, similar observations were made in colorectal cancer [7], [22].

To examine the contribution of differential Smad phosphorylation towards carcinogenesis in gastric cancer, we investigated P-Smad2/3C and P-Smad2/3L levels in 130 patients with gastric adenocarcinoma by immunohistochemistry (IHC). We analyzed potential correlations between different sites of phosphorylation of R-Smads and invasion, lymph node metastasis, differentiation, and stage of gastric cancer.

## Results

### Linker phosphorylation of Smad2/3 is not associated with gastric cancer

In contrast to previous findings from colorectal cancer and HCC [7], [8], [21], [22], we did not detect significant P-Smad2L and/or P-Smad3L positive staining in gastric tissues from patients with gastric cancer. Only 18 out of 130 patients showed p-Smad3L positive staining mainly in non-cancer cells. These results suggest that linker phosphorylation in serine sites of Smad2/3 is not critical in gastric cancer.

### Different features of P-Smad2C and P-Smad3C levels in gastric cancer

**Figure 1:**
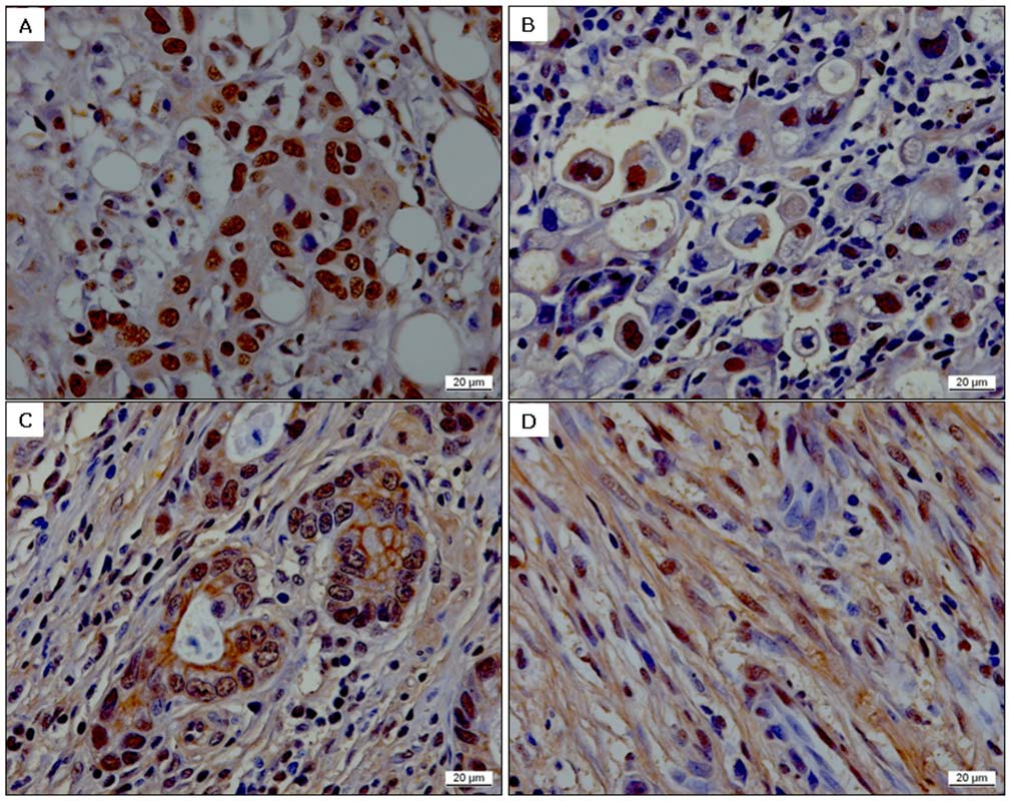
Patterns of P-Smad2C (**A**) and P-Smad3C (**B–D**) IHC staining in gastric cancer. (**B**) P-Smad3 nuclear staining; (**C**) P-Smad3 membrane/plasma staining; (**D**) P-Smad3 fibroblast staining.

118 out of 130 gastric tissues showed P-Smad2C positive staining, and 91 out of 130 gastric tissues were positive for P-Smad3C staining respectively. Distinct from P-Smad2C positive staining, which was only displaying in the nucleus of normal and cancer cells (**Fig. 1A**), P-Smad3C positive staining was demonstrated in the nucleus (59/130, **Fig. 1B**), at the membrane and/or in the cytoplasm (73/130, **Fig. 1C**) of gastric cancer cells. In addition, P-Smad3C was seen by positive staining in fibroblasts surrounding cancer cells (81/130 and 91/130, **Fig. 1D**).

### Reduced P-Smad2C remarkably correlates with tumor depth (T) and differentiation of cancer (G) in patients with gastric cancer

**Figure 2:**
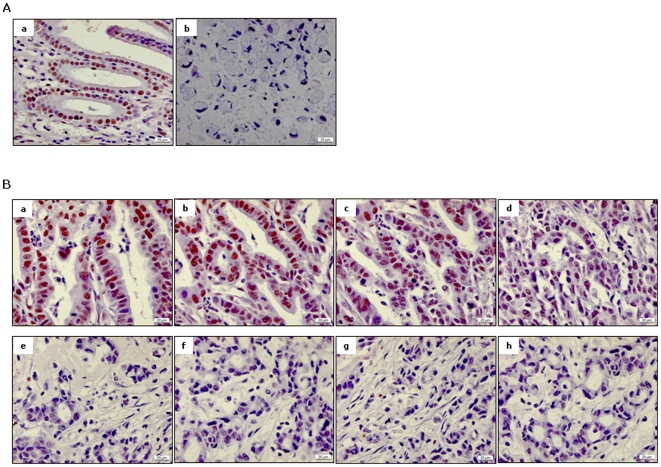
Loss of P-Smad2C in gastric cancer cells. (**A**) Immune positivity of P-Smad2C was significantly decreased in cancer tissues (*b*) when compared with surrounding normal tissues (*a*) in 77 out of 130 patients with gastric cancer. Representative pictures were taken from a patient with signet-ring cell carcinoma. (**B**) In tumor tissues, cancer cells with poor atypia (*e–h*) showed decreased P-Smad2C staining compared to those with a well-differentiated phenotype (*a–d*).

We measured P-Smad2C IHC staining not only in the tumor but also in surrounding non-tumor tissues from 130 patients with gastric cancer. P-Smad2C positive staining in either cancer or normal cells inversely correlated with differentiation of gastric cancer (both *p*<0.0001, **Table 1**). In addition, decreased P-Smad2C IHC score in cancer cells correlated with tumor depth (*p*<0.05, **Table 1**). P-Smad2C levels in normal tissue as well showed a potential inverse correlation with cancer invasion in these patients (*p* = 0.07, **Table 1**). The P-Smad2C IHC score was significantly decreased in cancer tissues compared with non-tumor tissues in 77 out of 130 patients with gastric cancer. Moreover, within the tumor tissues, cancer cells with poorly atypia showed decreased P-Smad2C staining, when compared with well-differentiated ones (**Fig. 2**). Kendall-tau correlation analysis revealed a potential correlation between reduction of P-Smad2C staining and the transition from normal to cancer cells in gastric cancer (*p* = 0.05, **Table 2**), suggesting that activation of Smad2 signaling is a critical suppressor of tumor formation and progression.

**Table 1:** Correlation between P-Smad2C/P-Smad3C/α-SMA positive cells and gastric cancer (n = 130).

	Size of tumor (T)		Lymph nodes (N)		Tumor differentiation (G)		TNM stage (S)	
	τ_B_	*p* value	τ_B_	*p* value	τ_B_	*p* value	τ_B_	*p* value
pS2C_cancer	−0.16	0.02	−0.08	0.25	−0.29	<.0001	−0.12	0.07
pS2C_normal	−0.13	0.07	−0.10	0.16	−0.30	<.0001	−0.12	0.09
pS3C_cancer	−0.07	0.35	−0.08	0.30	−0.05	0.56	−0.09	0.21
pS3C_normal	−0.08	0.31	−0.10	0.20	0.08	0.34	−0.09	0.25
pS3C_matrix	0.05	0.53	0.05	0.49	0.06	0.47	0.04	0.58
α-SMA	−0.03	0.66	−0.04	0.58	0.00	0.96	−0.04	0.52

Kendall-tau rank correlation coefficient.

**Table 2:** Correlation between P-Smad2C change (cancer cells versus normal tissues) and differentiation of gastric cancer (G) (n = 130).

pS2C (tumor vs.normal)	Down (%)	No change (%)	Up (%)	total
≤G2 (n = 36)	0,72	0,19	0,08	1,00
G3 (n = 79)	0,58	0,28	0,14	1,00
G4 (n = 14)	0,36	0,64	0,00	1,00
**τ_B_**				0.16
*p* value				0.05

Kendall-tau rank correlation coefficient.

### P-Smad3C is frequently found in cancer associated fibroblasts

P-Smad3C IHC score did not show a significant correlation with T/N/G/S of gastric cancer samples (**Table 1**). However, P-Smad3C positive staining often occurred in fibroblasts surrounding cancer cells in patients with gastric cancer (**Fig. 1**).

**Figure 3:**
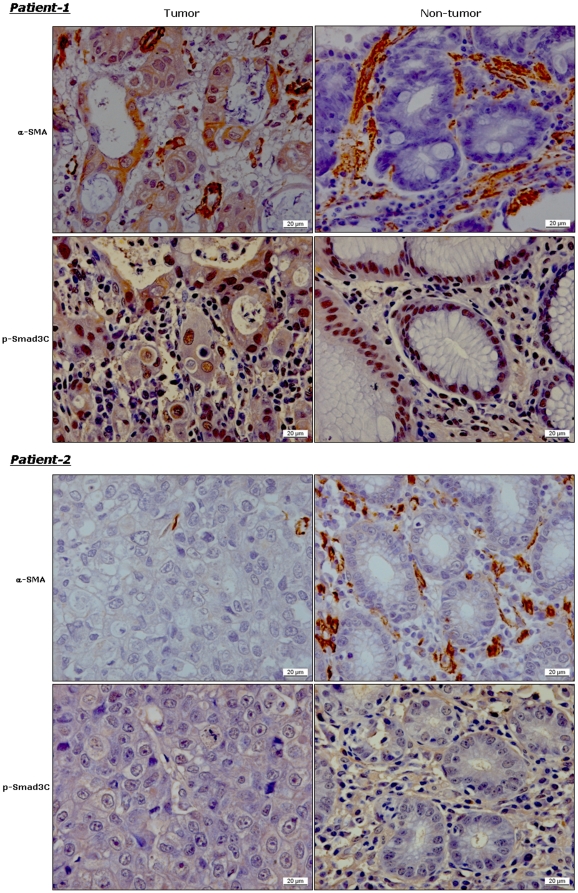
P-Smad3C nuclear staining demonstrates a positive correlation with α-SMA expression in gastric cancer cells, but not in surrounding non-tumor tissues. Representative pictures from two patients with gastric cancer show α-SMA and P-Smad3C staining in cancer area (tumor) and surrounding non-cancer tissues (non-tumor).

**Figure 4:**
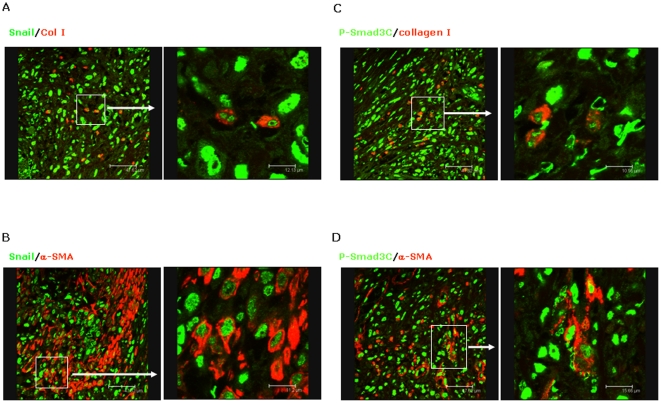
Gastric cancer cells express EMT markers, including Snail, collagen I and α-SMA. Confocal microscopy analysis demonstrates co-localization of Snail and collagen I (**A**)/α-SMA (**B**) in a representative patient with gastric cancer. The same patient in addition displays co-localization of P-Smad3C and collagen I (**C**)/α-SMA (**D**) in gastric cancer.

Cancer cells are of epithelial origin and do not express mesenchymal markers, e.g. α-SMA and collagen I. If cancer cells express mesenchymal markers, it is suggestive of epithelial-to-mesenchymal transition (EMT) [11]. In the present study, we found α-SMA positive staining in cancer cells in 54 of 130 patients with gastric cancer (e.g. patient-1 in **Fig. 3**). To clarify the potential link between p-Smad3C and EMT, we investigated the correlation between p-Smad3C staining (cancer and surrounding non-cancer tissue) and α-SMA positive staining in cancer cells. Kendall-tau rank correlation coefficients between p-Smad3C IHC scores in cancer/surrounding non-cancer tissue and α-SMA IHC scores in cancer are 0.14 (*p* = 0.08) and −0.09 (*p* = 0.99), respectively (**Table 3**). To confirm EMT in these patients, we performed co-staining for SNAIL, another specific marker for EMT, and collagen I/α-SMA in gastric tissues. Confocal microscopy revealed co-localization of SNAIL and collagen I (**Fig. 4A**)/α-SMA (**Fig. 4B**) in some gastric cancer cells. These α-SMA positive gastric cancer cells as well displayed P-Smad3C positive staining (**Fig. 4C**). Besides co-localization with α-SMA positive gastric cancer cells, confocal microscopy revealed that P-Smad3C co-localized with collagen I in gastric cancer cells (**Fig. 4D**). Further, P-Smad3C positive staining co-localized with α-SMA and collagen I not only in cancer cells, but also in fibroblasts (**Fig. 4C–4D**). These results suggest that P-Smad3C may contribute to EMT of gastric cancer in some patients.

**Table 3:** Correlation between P-Smad2C/P-Smad3C and α-SMA positive cells in gastric cancer (n = 130).

	pS3C_cancer		pS3C_normal	
	τ_B_	*p* value	τ_B_	*p* value
α-SMA	0.14	0.08	−0.09	0.99

Kendall-tau rank correlation coefficient.

### Loss of total Smad2 and Smad3 expression in patients with gastric cancer

**Figure 5:**
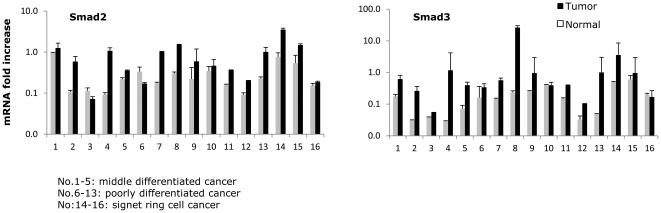
Real-time PCR was performed to determine mRNA expression of Smad2 (**A**) and Smad3 (**B**) in 16 gastric cancer tissues and surrounding non-tumor tissues (No. 1–5: middle differentiated cancer; No. 6–13: poorly differentiated cancer and No. 14–16: signet-ring cell cancer).

Besides measuring P-Smad2 in gastric cancer, we used real-time PCR to detect mRNA expression of Smad2 and Smad3 in gastric cancer and surrounding non-tumor tissues from 16 patients. 15 and 12 patients respectively demonstrated decreased mRNA expression of Smad2 and Smad3 in cancer tissues, when compared with surrounding normal tissues (**Fig. 5**). These results indicate that not only activated Smad2 but also the mRNA expression of total Smad2 is reduced during gastric carcinogenesis.

## Discussion

In colorectal adenocarcinoma and HCC, a shift in phosphorylation from carboxy-terminus to linker regions has been suggested as a critical event associated with the switch of TGF-β from a cancer suppressor to an oncogenic factor [8], [18], [19], [20], [21], [22]. In the present study, we examined this potential association in gastric cancer. We did not detect significant P-Smad2L and P-Smad3L levels in 130 patients with gastric cancer. 112 out of 130 patients did not show P-Smad2L or P-Smad3L staining in gastric cancer cells, suggesting that the proposed shift in phosphorylation of R-Smad may not contribute to TGF-β mediated carcinogenesis in this type of cancer. In contrast to P-Smad2/3L, both P-Smad2C and P-Smad3C positive staining was detected in most patients with gastric cancer. Kendall-tau rank correlation analysis revealed that P-Smad2C IHC score inversely correlates with depth of tumor (T) and differentiation of cancer (G).

The role of Smad2 during formation and progression of different cancer type remains controversial. Consistent with the current finding, previous studies on esophageal squamous cell carcinoma, breast cancer and colorectal cancer demonstrated that loss of Smad2 expression is correlated with tumor development and poor prognosis [23], [24], [25], [26]. Recently, Hoot and colleagues found that Smad2, but not Smad3, was frequently lost in 83 patients with skin squamous cell carcinoma. Further, mice with keratinocyte-specific Smad2 deletion displayed accelerated formation and malignant progression of chemically induced mouse skin tumors compared with wild type mice [27]. However, in a study of 52 patients with glioma, a positive P-Smad2 IHC score was reported to correlate with proliferation of gliomas and poor prognosis [28]. Prior to the present study, Shinto and colleagues measured P-Smad2C staining in 135 patients with gastric cancer. They found that the P-Smad2C levels were higher in poorly-differentiated cancer when compared with well-differentiated ones [29]. It is not clear yet why there are completely opposing results in gastric cancer.

In canonical TGF-β signaling, activated TGF-βRI usually induces carboxy-terminal phosphorylation of both Smad2 and Smad3. Activated Smad2 and Smad3 play different roles in cell growth, differentiation and other biological functions [30]. In the liver, Smad2 is critical in mediating hepatocyte growth and differentiation, whereas Smad3 plays a key effect in the morphological and functional maturation of hepatic stellate cells [31], [32]. In cancer, it is controversial whether disruption of the Smad3 gene contributes towards tumorigenesis. Zhu reported that Smad3-deficient mice develop colon carcinoma [33]. However, other studies using Smad3-deficient mice suggested that the loss of Smad3 alone is not sufficient to initiate tumorigenesis [34], [35], [36], [37]. In gastric cancer, Han found low levels of Smad3 in 3 out of 8 patients and in nine human gastric cancer cell lines [38]. Introduction of a Smad3 vector into gastric cell lines restored TGF-β responsiveness. Further, injection of Smad3-expressing gastric cells into nude mice showed delayed tumorigenesis when compared with controls, indicating a correlation of loss of Smad3 with carcinogenesis [38]. In the present study, we did not find a significant correlation between P-Smad3C staining in gastric cancer cells and cancer invasion, differentiation and stage. Distinct from P-Smad2C, which is only expressed in nuclei of cells, P-Smad3C positive staining is detectable in the nucleus, at the membrane and in the cytoplasm of gastric cancer cells. The biological relevance of different patterns of P-Smad3C staining in cancer cells is currently still unknown.

EMT of cancer cells is a prerequisite for progressing to advanced metastatic tumors [11]. It has been recognized that TGF-β is a major player in EMT. Smad3, but not Smad2, is a key mediator in TGF-β dependent EMT [39]. For instance, TGF-β fails to induce EMT in primary tubular epithelial cells derived from kidneys of Smad3-deficient mice [40]. Inhibition of TGF-β signalling by Ki26894, a TGF-βRI inhibitor, decreased invasion and EMT of scirrhous gastric cancer *in vitro* [41]. Our results show that part of gastric cancer cells express EMT markers, e.g. Snail, collagen I and α-SMA. Co-localization of P-Smad3C with α-SMA/collagen I is found in a substantial subset of patients. In addition, α-SMA positive staining in cancer cells has a potential correlation with P-Smad3C IHC score of cancer cells, but not surrounding non-cancer tissues. These results indicate that P-Smad3C might play a role in EMT of gastric cancer.

In Summary, the present study suggests that Smad2 is an important mediator to defend gastric cells from progressing towards poorly differentiated cancer. According to our results, Smad3 is not directly linked with characteristics of gastric cancer, however, our data indicated that it may play a role in cancer associated fibroblasts and EMT of gastric cancer cells.

## Methods

### Patients

Surgical specimens were examined from patients with primary gastric cancer at the Department of General Surgery, the First Affiliated Hospital, Medical School, Zhejiang University, China from 2003 to 2009. Gastric tissue specimen was collected when surgeries dissected the Gastric cancer. The collected tissues included tumor and surrounding non-tumor areas (tissues at least more than 5 cm far away from the tumor). A part of tissues were fixed with in 4% formaldehyde and embedded in paraffin for histology and IHC measurement. Remained fresh tissues were put into liquid nitrogen immediately for mRNA measurement. A total of 130 paired gastric tissues including cancer and surrounding non-tumor tissues were enrolled. Pathological diagnosis and classifications were estimated according to the tumor-node-metastasis (TNM) classification advocated by the International Union against Cancer (seventh edition) [42]. Basic characteristics of the enrolled patients are shown in **Table 4**. The study protocol conformed to the ethical guidelines of the Declaration of Helsinki (1975). The study was approved by the ethics committee of the First Affiliated Hospital, Medical School, Zhejiang University. Written informed consents were obtained from all participants involved in the study.

**Table 4:** Basic characteristics of 130 patients with gastric cancer.

	Number
**Age**	
<60	69
≥60	61
**Sex**	
M	88
F	42
**Location**	
Proximal end of stomach (1/3)	10
Gastric body (1/3)	22
Distal end of stomach (1/3)	59
More than 1/3 of the stomach involved	39
**Depth of tumor**	
Tis	3
T1	19
T2	32
T3	60
T4	16
**Lymph node metastasis (N)**	
No	47
N1	19
N2	29
N3	35
**Tumor differentiation (G)**	
Well diff.	22
Moderate Diff.	32
Poorly diff.	60
Undiff.	16
**TNM stage**	
0	3
Ia	16
Ib	13
IIa	27
IIb	6
IIIa	22
IIIb	27
IIIc	16

### Antibodies

Rabbit polyclonal antibodies against P-Smad2C (ser 465/467), P-Smad3C (ser 423/425), P-Smad2L (ser 250/255), and P-Smad3L (ser 208/213) were described in previous studies [43], [44]. Mouse anti-human α-SMA (M0851), polyclonal anti-SNAIL (ab-17732), and monoclonal anti-collagen I (C2456) antibodies were purchased from DAKO, Abcam and Sigma respectively.

### Immunohistochemistry (IHC)

After reviewing the H&E-stained slides from 130 enrolled patients with gastric cancer, representative paraffin blocks for each patient were selected for section. The slides were deparaffinised in xylene and rehydrated in a dilution series of graded ethanol to distilled water. Antigen retrieval was performed by microwave treatment in EDTA buffer (1 mMol, pH 8.0). The slides were then treated with 3% H_2_O_2_ for 30 min at room temperature. After washing with PBS three times, the slides were incubated with rabbit polyclonal antibody against P-Smad2C (1∶100), P-Smad3C (1∶100), P-Smad2L (1∶50), P-Smad3L (1∶50), and α-SMA (1∶200) respectively at 4°C overnight. The next day, the slides were washed with PBS three times. After washing, they were incubated with EnVision peroxidase (Dako) -labeled anti-rabbit or anti-mouse antibody for 1 h at room temperature. Peroxidase activity was detected with diaminobenzidine (DAB). The slides were counterstained with hematoxylin. Immunoreactivity was visualized under light microscopy.

For semiquantitative analysis, the stained tissue sections were assessed at a 4-point scale as follows: positive cell counts, grades 0–3 (0, no positive cells; 1, <25% positive cells; 2, 25%–50% positive cells; 3, >50% positive cells); intensity of positive staining, grades 1–3 (1, weak positive staining, usually yellow; 2, strong positive staining, usually brown; 3, very strong positive staining, usually deep brown to black). The final immune staining score was calculated as number×intensity.

### Immunofluorescence staining

The slides were deparaffinised in xylene and rehydrated in graded ethanol until distilled water. Then antigen retrieval was performed by microwave treatment in EDTA buffer (1 mMol, pH 8.0). The slides were then treated with 3%H_2_O_2_ for 30 min at room temperature. After washing with PBS, both primary antibodies, rabbit anti-P-Smad3C/Snail and mouse anti-α-SMA/collagen I, were applied at 4°C overnight. Then, secondary antibodies, Alexa633-rabbit anti-mouse immunoglobulin G and Alexa488-donkey anti-rabbit immunoglobulin G (Molecular Probes/Invitrogen, Karlsruhe, Germany), were applied for 30 min at room temperature. Samples were mounted using Dako-Cytomation Fluorescent Mounting Medium. The slides were inspected and pictures were taken on a confocal microscope (Leica, Heidelberg). Sections without primary antibodies were used as negative controls.

### Real time PCR

The total RNA was purified from gastric tissues with the High Pure RNA isolation kit (Roche Diagnostic GmbH, Mannheim, Germany) according to the manufacturer's protocol, and the concentration was measured spectrophotometrically. cDNA was synthesized from 1 µg of RNA with the Transcriptor First Strand cDNA synthesis kit (Roche Diagnostics). Quantitative real-time PCR was performed with an ABIPrism 7700 sequence detection system (Applied Biosystems) using a TaqMan universal PCR master mix No AmpErase UNG (part no. 4324018). The following gene expression assays were used: Smad2 (forward: CAAACCAGGTCTCTTGATGG; reverse: GAGGCGGAAGTTCTGTTAGG), Smad3 (forward: GGAGAAATGGTGCGAGAAGG; reverse: GAAGGCGAACTCACACAGC) and peptidylprolyl isomerase A (housekeeping gene; part. no. Mm02342429_g1). All reagents were purchased from Applied Biosystems. Samples were run in triplicate under the following conditions: initial denaturation for 2 minutes at 50°C and for 10 minutes at 95°C followed by 40 cycles of 15 s at 95°C and 1 minute at 60°C. The levels of gene expression in each sample were determined with the comparative cycle threshold method.

### Statistical Analysis

To evaluate association between selected immunohistological markers and tumor parameters, Kendall-tau rank correlation analysis was performed using SAS version 9.2 (Cary, NC, USA). A *p*<0.05 was considered statistically significant.
